# Prolactin and Prolactin Receptor Expression in Rat, Small Intestine, Intraepithelial Lymphocytes During Neonatal Developmen

**DOI:** 10.1155/2001/10175

**Published:** 2001

**Authors:** Sandra L. Urtishak, Elizabeth A. Mckenna, Andrea M. Mastro

**Affiliations:** 1 Department of Biochemistry and Molecular Biology The Pennsylvania State University 431 S. Frear University Park PA 16802 USA; 2 University of Pennsylvania School of Medicine Philadelphia PA 19104 USA; 3 University of Michigan Department of Pediatric Surgery Ann Arbor MI 48109 USA

**Keywords:** intestine, intraepithelial, lymphocytes, neonatal development, prolactin, rat, CD4 T cell

## Abstract

Intraepithelial lymphocytes (IEL) are specialized T cells found between the epithelial cells of the small intestine. Because of their location, IEL are the first lymphocytes to contact intestinal bacteria and food antigens. In the neonate, IEL may be the first cells of the immune system to interact with milk-borne hormones including prolactin (PRL). PRL, an endocrine hormone abundant in breast milk, interacts with cells through surface receptors. PRL has been shown to function as an immunoregulator and may affect the development of the newborn's immune system. To determine if PRL plays a role in IEL development, small intestine IEL from rats of various ages were examined for the presence of surface prolactin receptor (PRL-R) and several lymphoid markers by flow cytometry. Between birth and 96 days of age about 80% of IEL were found to express PRL-R. These same cells also expressed the mRNA for PRL. Additionally, all of the IEL subpopulations examined were found to express PRL-R. Analysis of the normal development of rat IEL revealed an age related increase in total IEL, CD4 positive cells as well as a peak in interleukin-2 receptor (IL-2R) expression at weaning. In summary, the results indicate that IEL express PRL and PRL-R. In addition, an activation marker, IL-2R, changes in expression during neonatal development.


*Developmental Immunology*, 2001, Vol. 8(3-4), pp. 319-330
Reprints available directly from the publisher
Photocopying permitted by license only

© 2001 OPA (Overseas Publishers Association)
N.V. Published by license under
the Harwood Academic Publishers imprint,
part of The Gordon and Breach Publishing Group,
a member of the Taylor and Francis Group

### Prolactin and Prolactin Receptor Expression in Rat, Small Intestine, Intraepithelial Lymphocytes During Neonatal Developmen*

SANDRA L. URTISHAK†, ELIZABETH A. McKENNA‡ and ANDREA M. MASTRO¶

*Department of Biochemistry and Molecular Biology, The Pennsylvania State University, 431 S. Frear, University Park, PA 16802, USA*

Intraepithelial lymphocytes (IEL) are specialized T cells found between the epithelial cells of the small intestine. Because of their location, IEL are the first lymphocytes to contact intestinal bacteria and food antigens. In the neonate, IEL may be the first cells of the immune system to interact with milk-borne hormones including prolactin (PRL). PRL, an endocrine hormone abundant in breast milk, interacts with cells through surface receptors. PRL has been shown to function as an immunoregulator and may affect the development of the newborn's immune system. To determine if PRL plays a role in IEL development, small intestine IEL from rats of various ages were examined for the presence of surface prolactin receptor (PRL-R) and several lymphoid markers by flow cytometry. Between birth and 96 days of age about 80% of IEL were found to express PRL-R. These same cells also expressed the mRNA for PRL. Additionally, all of the IEL subpopulations examined were found to express PRL-R. Analysis of the normal development of rat IEL revealed an age related increase in total IEL, CD4 positive cells as well as a peak in interleukin-2 receptor (IL-2R) expression at weaning. In summary, the results indicate that IEL express PRL and PRL-R. In addition, an activation marker, IL-2R, changes in expression during neonatal development.

*Keywords:* intestine, intraepithelial, lymphocytes, neonatal development, prolactin, rat, CD4 T cell

### INTRODUCTION

Evidence suggests that prolactin (PRL), a peptide hormone secreted by the anterior pituitary, plays an important role in immunoregulation as both hypoprolactinemia and hyperprolactinemia lead to a compromised immune system (Reber, 1993). Additionally, PRL induces the alpha chain of the interleukin-2 receptor (IL-2), a lymphocyte activation marker, on splenocytes (Mukherjee et al., 1990); and activated lymphocytes synthesize and secrete PRL (Pellegrini et al., 1992). The PRL receptor (PRL-R) has been found on circulating leukocytes (Russell et al., 1984) as well as on splenocytes and thymocytes (Viselli and Mastro, 1993; Koh and Phillips, 1993).

* Submitted in partial fulfillment of an honors degree in Biochemistry and Molecular Biology for Sandra L. Urtishak and a M.S. degree in Biochemistry and Molecular Biology for Elizabeth A. McKenna.

† Present address, University of Pennsylvania School of Medicine, Philadelphia, PA 19104.
‡ Present address, University of Michigan, Department of Pediatric Surgery, Ann Arbor, MI 48109.
¶ Corresponding author: Andrea M. Mastro, 431 S. Frear, University Park, PA 16802, USA. phone: (814) 863–0152, fax: (814) 863–7024, e-mail: a36@psu.edu

PRL passes from the circulation of lactating rats to the milk. The concentration and forms of PRL found in milk suggest that it may be important for the neonate as well as for the mammary gland. In fact, maternal milk PRL has been found to pass into the plasma of suckling rats (Whitworth and Grosvenor, 1978). Its role in the neonate is not clear but there is evidence that PRL can regulate neonatal pituitary development (Porter and Frawley, 1993), as well as the development of the neonatal immune system (Grove et al. 1991). In a previous study it was found that milk ingestion during the first 7 hr. of life decreased the percentage of splenocytes and thymocytes expressing PRL-R (Gunes and Mastro, 1996). Furthermore, splenocytes and thymocytes from neonatal rats ingesting PRL-poor milk showed an accelerated and increased response to mitogens when tested *ex vivo* (Grove et al., 1991).

Because of their location, intraepithelial lymphocytes (IEL) present in the small intestine may represent the first interaction between milk-borne PRL and the immune system. With the exception of one study that identified mRNA for PRL and PRL-R in an enriched mouse intestinal IEL preparation by PCR (Nagano et al., 1995), the presence and role of PRL-R on IEL has not been examined. In this study, our aim was to determine if IEL expressed PRL and PRL-R, if so what subpopulations were positive, and if this expression changed during development or with milk ingestion.

### RESULTS

### Isolation and Characterization of Rat IEL

The number of IEL recovered per intestine increased with the age of the animal (Fig. 1). At birth $< 1 \times 10^6$ IEL were recovered per intestine but later in the neonatal period the value increased to about $3 \times 10^6$. The number continued to increase after weaning and reached adult levels of about $13 \times 10^6$ per intestine after about 50 days.

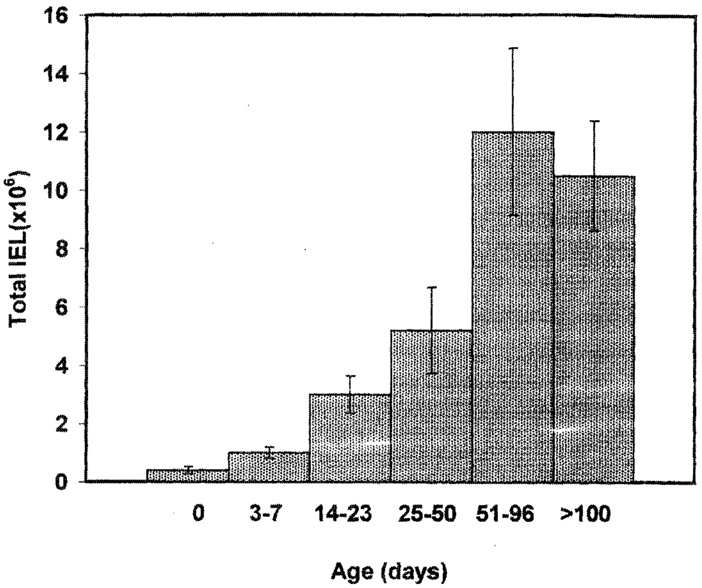

FIGURE 1 Total IEL recovered at various ages. IEL were isolated from the small intestines of Sprague Dawley rats following the procedure of Kearsey and Studynk (1996) as described in the methods section. Shown are the average number of IEL ± S.E.M. per intestine. The number of intestines used per isolation varied with age (see Methods). There were 4 isolations for 0 days and 10 isolations for 3-7 days; 5 isolations for 14–23 days, 6 isolations each for 25–50 days and 51–96 days and 10 isolations for > 100 days

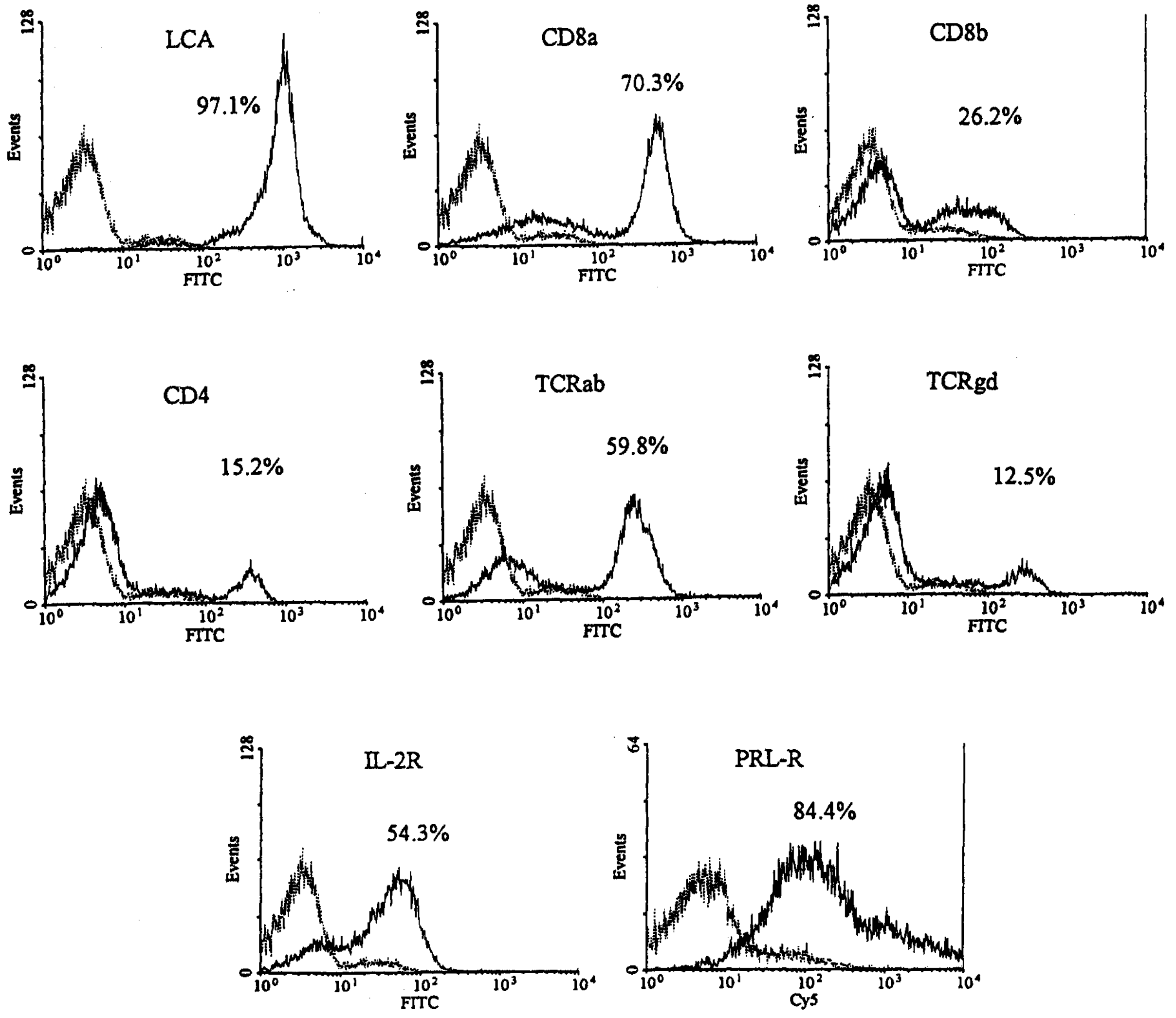

FIGURE 2 Flow cytometric characterization of IEL. IEL were isolated from the small intestine of Sprague Dawley rats and analyzed by flow cytometry on a Coulter XL2 flow cytometer with Elite software. LCA expression was used to gate the lymphocyte population. Representative data from a 96 day male rat are shown. Dashed lines show the negative controls and solid lines show staining with the various lymphoid markers and PRL-R. Values are the percentages of positively stained cells

In order to characterize the isolated cells and to verify that they were indeed IEL, phenotyping was carried out. Small intestine IEL from Sprague-Dawley rats at ages between 0 and 270 days were examined by flow cytometry (Fig. 2). Depending on the particular preparation, between 50% and 90% of the isolated cells expressed leukocyte common antigen (LCA). Since all lymphocytes express LCA and epithelial cells do not, LCA expression was used as the standard for flow cytometric gating. At all ages, an average of 92.9% ± 5.6% of the gated lymphocyte population, expressed leukocyte common antigen (LCA) (range 81.4 – 99.4%; n=29) (Table I, Fig. 2). Of the LCA positive cells, 66.6% were also positive for CD8. IEL expressed either the CD8αα homodimer (43.9% ± 8.7%) or the CD8αβ heterodimer (19.3%± 9.9%). TCRαβ cells were 56.4% ± 7.7% of the total LCA positive cells while TCRγδ

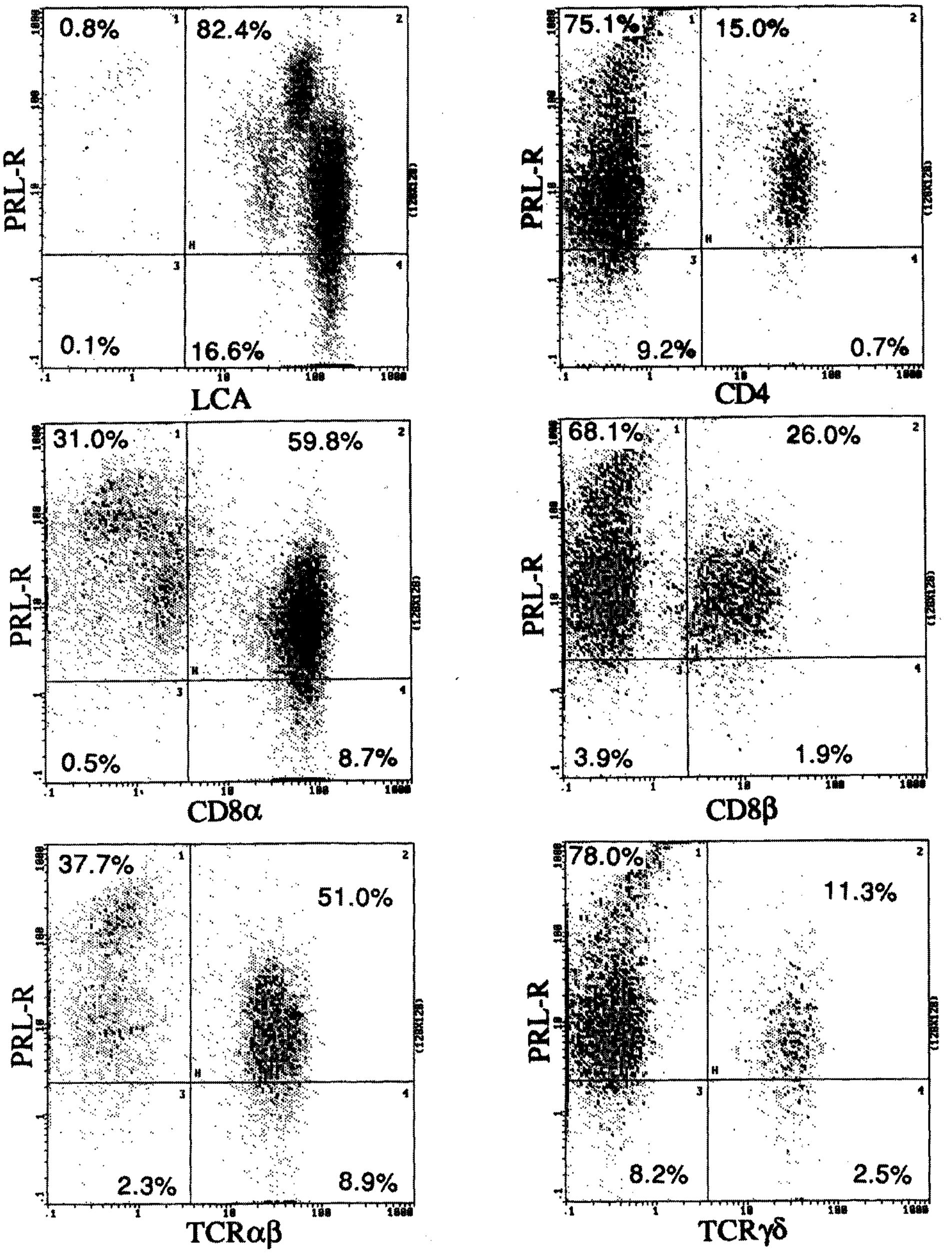

FIGURE 3 Dual labeling of IEL for PRL-R and lymphoid markers. IEL were isolated from the small intestine of Sprague Dawley rats and analyzed by flow cytometry on a Coulter XL2 flow cytometer with Elite software as described in the methods section. LCA expression was used to gate the lymphocyte population. Data from a 96 day male rat are shown

(8.9% ± 2.6%) expression was lower (Table I, Fig. 2). CD4 expression was also detected in IEL but the percentage of IEL expressing CD4 varied with age (see Fig. 4).

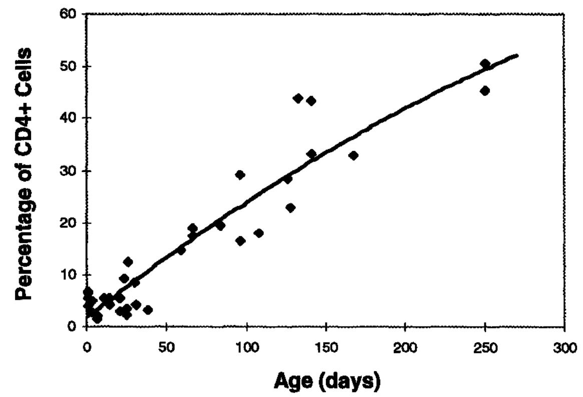

FIGURE 4 Change in CD4 expression with age. IEL were isolated from Sprague-Dawley rats between 0 and 250 days old. After staining with a monoclonal antibody to CD4, IEL were analyzed by flow cytometry. First degree polynomial regression was used to generate the best fit line

TABLE I Phenotype of rat small intestine IEL

| *Surface Marker* | *Percentage of Cells*[a] |
|---|---|
| Total CD8 | 66.6 ± 12.8 |
| CD8αα | 43.9 ± 8.7 |
| CD8αβ | 19.3 ± 9.9 |
| TCRαβ | 56.4 ± 7.7 |
| TCRγδ | 8.9 ± 2.6 |
| PRL-R | 82.3 ± 6.4 |

Isolated IEL were phenotyped using antibodies and flow cytometric analysis as described in the methods section.

a. The percentages of IEL expressing lymphoid markers were normalized by setting LCA expression to 100% (range 81.4–99.4%). For LCA the n value = 29. For the other markers n=20 except PRL-R where n=12.

### PRL-R Expression

The majority (82.3% ± 6.4%) of LCA positive IEL were found to express PRL-R (Table I, Fig. 2). In dual labeling experiments, PRL-R expression was detected on cells of each of the IEL sub-populations tested (Fig. 3). Approximately 85% of TCRαβ IEL (84.8% ± 7.7%) and 84% of TCRγδ IEL (84.2% ± 2.6%) expressed PRL-R. PRL-R were also found in approximately 86% of the CD8 positive IEL (85.8% ± 7.9%), 88% of the CD4 positive IEL (88.4% ± 7.7%), and 89% of IEL expressing the CD8β chain (89.1% ± 3.0%) (Fig. 3).

We also noted the presence of a subpopulation of IEL cells that stained about 10 times more brightly for PRL receptor and about 4 fold less brightly for LCA than the bulk of the IEL (Fig. 3). These cells were $LCA^+$, $CD4^-$, $CD8\alpha^-$, $CD8\beta^-$, $TCR\alpha\beta^-$, $TCR\gamma\delta^-$. This population made up about 15–20% of the total $LCA^+$ cells. Based on backgating and forward and side scatter, these cells are not dead cells or epithelial cell contaminants. At this time these cells have not been further characterized.

In order to determine if IEL expression of PRL-R was affected by ingestion of milk PRL, we examined IEL from a litter of newborn rats half of which were permitted to suckle and half of which were kept warm but not permitted to ingest milk. We found that PRL-R expression was present on IEL at 10 hr. of age regardless of whether milk had been ingested. We also examined PRL-R throughout the neonatal period and into adulthood. Although the percentage of IEL expressing PRL-R did not change during development, the relative intensity of PRL-R did show a trend to increase with age. The intensity of PRL-R expression was highly variable, but on average a minimal at 14 days of age and a maximal at 66 days of age.

### Other Age Associated Changes in IEL

In the course of examining PRL-R expression, two other age associated changes in IEL were identified. First, the percentage of IEL expressing CD4 greatly increased with age. CD4 expression averaged 4.6% for rats 21 days and younger. However, by 250 days of age 45–50% of IEL expressed CD4 (Fig. 4). Dual labeling indicated that the increase in CD4 positive cells was due to an increase in dual CD4 positive CD8αα positive IEL. In two animals (141 days of age) approximately 90% (89%, 92%) of the dual labeled cells were of the CD8αα type.

Second, expression of the α chain of IL-2R was found to vary with age (Fig. 5). An antibody to the α chain of the IL-2R labeled about 40% of IEL from 10 hr. old rats. IL-2R expression increased to 70% – 75% between 7 and 21 days of age and to approximately

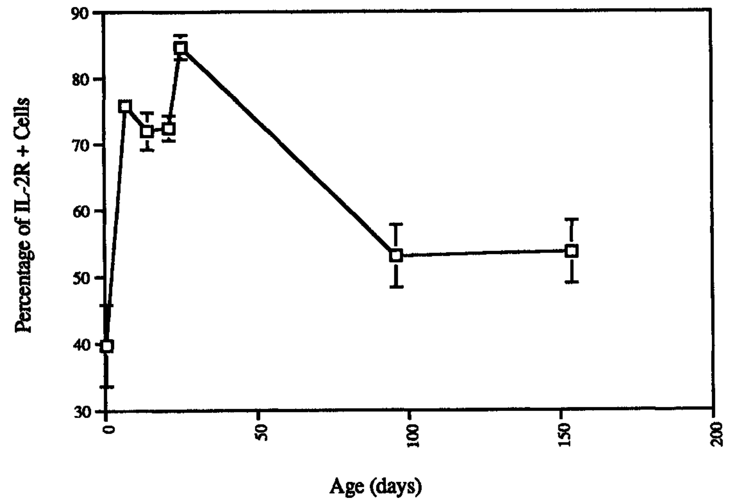

FIGURE 5 Change in IL-2R expression with age. IEL from Sprague Dawley rats were isolated and analyzed by flow cytometry after staining with a monoclonal antibody to the chain of the IL-2 receptor. Values are shown as percentages ± one standard deviation. n=2 for all ages. One way ANOVA indicated that IL-2R expression at times < 50 days varied significantly from that at > 50 days ($p < 0.05$)

85% at 25 days of age. However, by 96 and 154 days of age, IL-2R expression had dropped to about 50%.

### PRL Expression

Because other lymphocytes have been shown to express PRL as well as PRL receptor, we asked if IEL did the same. PRL mRNA was detected in all samples of rat IEL tested from 0 to 150 days of age. The relative PRL mRNA levels for animals age 0, 1, 7, 14, 21, 28 and 150 days were 2.86, 2.34, 0.83, 22.34, 3.91, 1.00 and 2.23 respectively. This experiment was carried out again with animals from 3, 14, 21, 28 to 52 days. The relative PRL mRNA levels were 2.52, 14.52, 2.60, 1.00 and 3.07. Day 28 values were set equal to 1.00 in both series for comparison. With the exception of a possible increase at day 14, the IEL PRL mRNA levels remained relatively constant from birth to 150 days of age.

### DISCUSSION

As reported previously for PVG rats (Lyscom and Brueton, 1983), IEL can be seen in the small intestine at birth. However, they increased in numbers throughout the neonatal period and reached adult levels somewhere around 2 months of age. On average about 10–15 × $10^6$ IEL can be isolated per small intestine of an adult rat following the procedure of Kearsey and Stadnyk (1996).

IEL isolated from the small intestine of rats between 0 and 270 days were found to be predominately (~70%) CD8 positive T cells. Approximately 56% of IEL expressed the αβ TCR and 9% expressed the γδ TCR. These values were very similar to those of Kearsey and Stadnyk (1996), whose isolation procedure we followed. They found IEL of Lewis rats to be approximately 70% CD8 positive, 75% TCRαβ positive, and 9% TCRγδ T cells. The data will also agree with results of a recently published procedures by Todd et al. (1999).

Kühnlein et al. (1995) found a similar percentage of TCRγδ postitive T cells in Wistar rats. Human IEL are approximately 10% TCRγδ positive similar to the rat. In contrast, mice IEL are 20–80% TCRγδ positive. Steege et al. (1997) reported 50% TCRγδ positive IEL by 20 days of age in mice. Since TCRγδ positive and CD8αα positive T cells are rare in other lymphocyte populations, TCRγδ and CD8αα IEL

may develop in the intestine from T cell precursors (Lefrancois, 1994). In Sprague Dawley rats about 70% of the CD8 positive IEL expressed the CD8αα homodimer and the remaining 30% expressed the CD8αβ heterodimer. Like rat IEL, IEL from mice and humans are predominately CD8 positive (90% in mice, 80% in human) and the majority of CD8 positive IEL express the CD8αα homodimer in these species (Lefrancois, 1994).

Based on our previous findings concerning PRL-R expression by thymocytes and splenocytes, we were interested in determining if IEL expressed PRL-R and if this expression changed during development. More than 80% of IEL expressed PRL-R, indicating that IEL are capable of interacting with PRL. In contrast to IEL only 17% of splenocytes (Viselli and Mastro, 1993) and 10% of thymocytes (Gunes and Mastro, 1996) express PRL-R in rats. The high percentage of IEL expressing PRL-R indicate that PRL may be particularly important in the regulation of IEL. PRL-R was identified on both TCRαβ positive and TCRγδ positive IEL as well as on CD8αβ positive, CD8αα positive, and CD4 positive IEL. The presence of PRL-R on all major IEL subpopulations suggests that PRL plays a role in the regulation of both thymus dependent and thymus independent IEL. Studies underway in our laboratory indicate that both long and short forms of PRL-R mRNA can be detected in flow cytometrically sorted rat IEL.

Although the percentage of IEL expressing PRL-R did not vary with age, the intensity of PRL-R did increase with age. The intensity of PRL-R expression is directly related to the number of PRL-R per cell. However, there was a lot of variation and these experiments will need to be verified.

We originally studied IEL from rats of different ages to determine how PRL-R might change with antigen exposure. While the percentages of cells expressing PRL-R did not vary, the percentage of IEL from Sprague Dawley rats expressing CD4 was found to increase with age from about 5% for animals less than 22 days to more than 45% at 250 days. By histochemical methods Lyscom and Brueton (1983) were not able to detect $CD4^+$ rat IEL before 2 weeks of age. However, by flow cytometry this small population at birth was clearly seen. In a flow cytometric study with mouse small intestinal IEL, Steege et al. (1997) reported that the percentage of $CD4^+$ cells increased from 11% on day 20 to 30% on day 30.

In the present study, the increase in CD4 positive IEL was not accompanied by a decrease in CD8 positive IEL. Therefore, the increase in CD4 positive IEL is most likely due to an increase in CD4/CD8 dual positive IEL. Takimoto et al. (1992) reported an increase in CD4/CD8 dual positive IEL between 4 and 30 weeks in several other rat strains. In the present study, dual labeling revealed that CD4 was found primarily on CD8αα. Immature CD4/CD8 double positive T cells are found in the thymus but are rare in circulating lymphocyte populations and in most secondary lymphoid organs. Thus, the CD4/CD8αα double positive IEL represent a population characteristic of the intraepithelial immune system.

The α chain of IL-2R also showed an age associated change. As a marker of lymphocyte activation, IL-2R is normally only present on activated lymphocytes. An increase in IL-2R expression by IEL was seen between 0 and 25 days of age. The percentage of IEL expressing IL-2R peaked at 25 days and then dropped to about 60% of the peak value by 96 days. The peak in percentage of activated IEL occurred just after weaning. A peak in intestinal immune system activity at weaning age has also been reported in mesenteric lymph nodes and mast cells of the intestine (Thompson et al., 1996). Steege et al. (1997) reported similar peaks in IEL activity at about the time of weaning. Since young rats begin eating solid food before weaning, the increase in IEL activation is probably not caused solely by foreign antigens in the solid food. However, it may reflect the removal of exposure to hormone such as PRL and other factors in milk. The greater percentage of IL-2R positive IEL in milk-deprived rats supports the hypothesis that a milk-borne factor suppresses IEL activation. Previous studies have found that splenocytes and thymocytes from neonatal rats fed PRL-poor milk showed an increased response to mitogens (Grove et al., 1991). Examination of IEL from neonates fed PRL-poor milk could determine if IEL activation is specifically related to PRL ingestion.

Rat somatotraphs do not produce PRL before day 5 (Hoeffler et al., 1985). Therefore the only source of PRL in the newborn is milk (Kacsoh et al., 1993) or locally produced PR1. While PRL is produced primarily by the pituitary, several other cell types express it as well, e.g. peripheral blood mononuclear cells (Sabharwal et al., 1992), splenocytes (Shah et al., 1991) and thymocytes (Montogery et al., 1992). PRL's function in these cells is not known, but it increases following cell activation (Montgomery et al., 1992). In this study we found PRL mRNA to be expressed by IEL from birth to relatively old age for the rat. We do not have evidence that the protein is made and secreted. However, Stevens and Show (1982) previously demonstrated the presence of immunoreactive presence in the small intestine by immunohistochemistry. They did not determine which cells in the small intestine were the source of the PRL. Our data suggests that the IEL are a source of intestinal PRL.

The characteristic phenotype of IEL may make these cells particularly well suited to immunological defense in the intestine. However, there is still little known about the development and *in vivo* function of IEL. Our finding of both PRL and PRL-R expression on the majority of IEL suggests that PRL plays some role in the regulation of all IEL subpopulations. Additionally, early milk ingestion may help regulate the activation of IEL.

Recent results from knockout mice lacking PRL-R (Ormandy et al., 1997) or PRL (Horseman et al., 1997) indicate that PRL is not necessary for life nor is it apparently essential for the gross development of the immune system. However, PRL like many cytokines may act reduntantly, and its role as an immunomodulator may be difficult to assess.

### MATERIALS AND METHODS

### Animals

Sprague-Dawley rats (Harlan Laboratories, Indianapolis, IN) were housed in a conventional animal facility at 20–22 °C on a 14 hr. light, 10 hr. dark cycle with free access to food and water. Pups remained with their mother until weaning at 21 days of age. All procedures were approved by the Animal Use and Care Committee of the Pennsylvania State University.

### Isolation of IEL

IEL were isolated from the small intestine of rats 14 days and older by a modified version of the procedure of Kearsey and Stadnyk (1996). Briefly, the small intestine from 1 cm below the stomach to 1 cm above the large intestine was removed and divided in half. Each half was flushed with PBS, everted, filled with PBS and ligated at the ends with surgical thread. The intestinal segments were placed in ice cold PBS containing 2 mM dithiothreitol (DTT) and vortexed for 10 s. Intestine segments were then placed in complete RPMI [5% v/v fetal bovine serum (FBS), 2 mM L-glutamine, 10 mM HEPES buffer, 50 U/ml penicillin, 50 μg/ml streptomycin] and vortexed for six bursts of 15 s at the highest speed. Intestine segments were discarded and the remaining cell suspension was passed through two layers of cheesecloth. 1X Percoll [10% (v/v), 10X PBS, 90% (v/v) Percoll (Sigma)] was added to the cell suspensions to give 30% (v/v) Percoll. Tubes were centrifuged at 500–550 × g for 15 min. at room temperature. Cells passing through the 30% Percoll were resuspended in 45 % (v/v) 1X Percoll in complete RPMI-1640 medium and layered above an equal volume of 75% (v/v) 1X Percoll in complete RPMI-1640 medium. The gradients were centrifuged at 500–550 × g for 30 min. at room temperature. Cells were recovered from the 45–75% interface and washed three times in complete RPMI-medium. Cell viability was consistently determined to be greater than 95% by trypan blue exclusion. For animals 50 days and older, recovery of IEL averaged 13 × $10^6$ cells per intestine. IEL recovery averaged 5.7 × $10^6$ cells per intestine for rats between 25 and 50 days of age.

For animals younger than 14 days of age where intestines were too small to be inverted, the intestine was removed, flushed with PBS, opened longitudinally, and cut into 1 cm sections. The sections were placed in PBS with 2 mM DTT and the cells were isolated as described for the larger segments. For rats between 11 and 23 days average recovery of IEL was $3.7 \times 10^6$ per intestine. An average recovery of $0.8 \times 10^6$ cells per intestine was obtained for animals between 0 and 7 days of age.

### Antibodies

The following antibodies were used for flow cytometric analysis: MRC OX-1 (anti-leukocyte common antigen LCA), MRC OX-8 (anti-CD8 α-chain), W3/25 (anti-CD4), OX-39 (anti-IL-2 receptor), [purchased from Serotec Inc., Raleigh, NC]; R73 and Rphycoerythrin (PE) conjugated R73 (anti-αβ T cell receptor), V65 and R-PE conjugated V65 (anti-γδ T cell receptor), R-PE conjugated OX-8 (anti-CD8 α-chain), fluorescein isothiocyanate (FITC) conjugated 341 (anti-CD8 β-chain), MRC OX-33 (anti-CD45, B cell form), PE and FITC conjugated 107.3 (Mouse $IgG_1$, κ isotype control) [purchased from Pharmingen, San Diego, CA]. FITC conjugated rabbit anti-mouse IgG HpositiveL and biotin conjugated donkey anti-rabbit IgG HpositiveL were purchased from Jackson Laboratories (West Grove, PA). R-PE Cy5 conjugated Streptavidin was purchased from DAKO (Carpinteria, CA).

A polyclonal antibody to PRL-R was a generous gift from Dr. Kurt Ebner, University of Kansas. This antiserum has been characterized in our laboratory (Viselli and Mastro, 1993) and in that of Kurt Ebner (Ebner et al., 1989, Bajpai et al., 1991). Ebner et al. (1989) showed that the anti-PRL receptor serum stimulated proliferation of Nb2 cells, a PRL dependent, PRL receptor positive cell line, presumably because binding of the antibody to the receptor mimics binding of PRL. In addition, binding of the anti-PRL receptor antibody to Nb2 cells, detected by use of a fluorescently labeled secondary antibody and fluorescence microscopy, decreased when the cell PRL receptors were first downregulated in the presence of PRL (Ebner et al., 1989). The antibody also inhibits PRL induced DNA synthesis in a normal splenocyte assay for PRL (Viselli and Mastro, 1993). This inhibitory activity was removed by pre-incubation with PRL receptor positive Nb2 cells; while antiserum adsorbed with rat red blood cells retained its ability to inhibit proliferation. Normal rabbit serum had no affect on DNA synthesis.

For flow cytometric analysis, the antiserum to PRL receptor was compared with normal rabbit serum and with irrelevant polyclonal rabbit serum to myosin light chain. Only between 1 and 2% of the cells were positive with either control. In addition, dilution of the anti-PRL serum with irrelevant rabbit serum (anti myosin light chain) did not change the percentage of positive lymphocytes. Pre-adsorption of the antiserum with PRL receptor positive Nb2 cells decreased staining to lymphocytes to near background levels (Viselli and Mastro, 1993).

### Immunofluorescent Staining and Analysis

All antibodies were diluted in PBS with 5% calf serum, 2% goat serum and 0.1% $NaN_3$ and used at saturating concentrations of 1 μg/1 $x10^6$ cells. For single color immunofluorescence, cells were incubated with diluted primary antibodies for 25 min at 4°C. Cells were washed twice in PBS with 5% calf serum, 2% goat serum and 0.1% $NaN_3$ and incubated with the secondary, FITC conjugated antibody. The cells were washed twice as described and stored in 1% formaldehyde in PBS in the dark at 4°C until analysis, usually within 2 days.

For two color immunofluorescent analysis, cells were incubated with saturating concentrations of anti-PRL-R serum and monoclonal antibodies for 25 min. at 4°C. After two washes in PBS with 5% calf serum, 2 % goat serum and 0.1 % $NaN_3$, cells were incubated with FITC rabbit conjugated anti-mouse IgG HpositiveL and biotin conjugated donkey anti-rabbit IgG HpositiveL for 25 min. at 4°C. Cells were washed twice as above and incubated with Cy5 conjugated streptavidin diluted 1/10 (50 μl / $0.25 \times 10^6$ cells). After two washes, cells were stored in 1% formaldehyde in PBS in the dark at 4°C until analysis.

Samples were analyzed with a Coulter XL2 flow cytometer. Tight forward and side scatter gates were set using LCA expression to eliminate contaminating epithelial and presumed dead cells. Backgating was used to verify that the various populations were contained within that gate. In double labeling experiments, compensation was set using the individually labeled antibodies with each fluorochrome.

When primary control antibodies of the same isotype as the mouse monoclonal antibodies were used, less that 3% of the gated cells were positive. The same low background labeling was seen when the secondary antibodies, FITC conjugated rabbit anti-mouse IgG and biotin conjugated anti-Rabbit IgG with R-PEcy5 conjugated streptavidin, were used in the absence of the primary antibody. These background values were subtracted from the experimental values. The percentage of cells expressing the CD8αα homodimer was determined by subtracting the percentage of cells that were labeled with the antibody to CD8αβ.

### Isolation of RNA and quantitative PCR

Isolated IEL were incubated with antibodies to CD8α and to LCA and FITC conjugated rabbit anti-mouse antibodies and sorted based on FITC fluorescence and lymphocyte-indicative forward angle and side angle light scatter using either a Coulter EPICS 753 (experiment 1) or a Coulter EPIC ELITE (experiment 2) (Coulter, Fullerton, CA) flow cytometer. A sample of the sorted cells was re-run through the flow cytometer to confirm purity. Post-sort IEL were 99.6 and 99.7% pure.

Up to $1.5 \times 10^6$ of the sorted IEL were lysed by vortexing in 400 $\mu\ell$ of guanidinium isothiocyanate (Gibco BRL, Grand Island, NY), 10% β-mercaptoethanol (Sigma Molecular Biology Grade, St. Louis) and stored at – 80 °C.

Frozen cell lysates were thawed at RT, incubated in a 37°C water bath for 10 minutes, and total RNA recovered using the Gibco BRL GlassMAX Total RNA Isolation Spin Cartridge System (Gibco BRL, Grand Island, NY) (experiment 1) or the QIAGEN RNeasy system and QIAshredder (QIAGEN Inc., Chatsworth, CA) (experiment 2) and stored at – 80 °C in DEPC-treated $H_2O$. Total RNA was quantified via UV absorbance readings at 260 nm derived from a scan of absorbances ranging from 310 to 210 nm in a UV/Vis spectrophotometer (DU-40, Beckman Instruments Inc., Fullerton, CA).

Contaminating genomic DNA contained in some samples was removed by treatment with Dnase I (Gibco BRL, Grand Island, NY) under conditions described in the GlassMAX Total RNA Isolation Spin Cartridge System manual (Gibco BRL, Grand Island, NY).

DNA primers for PCR were Synthesized by the Penn State Nucleic Acid Facility (University Park, PA) on an Oligo 1000M DNA Synthesizer (Beckman Instruments Inc, Fullerton, CA). PRL forward primer, 5′ GGA AGT GTG GTC CCA GTG GT 3′, anneals to nucleotides 25–44. PRL reverse primer, 5′ TGG CAG GGT CTG CAC ATT T3′, anneals to nucleotides 141–123. PRL fluorogenic probe, 5′ FAM-AAC AGC CAG GTG TCA GCC CGG AAA G-TAM 3′(synthesized by Synthetic Genetics, San Diego), anneals to PRL cDNA nucleotides 55–79. (PRL cDNA sequence as numbered in Cooke et al., 1980).

All quantitative RT-PCR reactions were run by the Penn State Nucleic Acid Facility on an ABI PRISM 7700 Sequence Detector (Perkin-Elmer, Foster City, CA). For each reverse transcription reaction, total RNA samples were incubated 5 minutes at 65°C, then placed on ice. 2.5 ul (experiment 1:1 to 2 ng; experiment 2:25 to 80 ng) of total RNA were added to 17 ul of reaction mixture containing the following: 1 U RNase Inhibitor; 1X Buffer; 5 mM $MgCl_2$; 500 uM of each dNTP, A, C, G, T; 750 nM reverse primer; 22 U Reverse Transcriptase. Each complete reaction mixture was incubated 1 hour at 42°C, 5 minutes at 72°C, 2 minutes at 25°C.

Standard controls used by the Penn State Nucleic Acid Facility (University Park, PA) for RT-PCR of rat target mRNA were run for each sample of siIEL total RNA. The control reactions measured RNA encoding rat ribsomal phosphoprotein PO (RPPO) which is constitutively expressed in all rat cells. The primers and probe sequences for RPPO are in file with the Nucleic Acid Facility.

Quantitative RT-PCR data analysis was performed using the Comparative $C_t$ Method described in the ABI PRISM 7700 Sequence Detection User Bulletin #2, PE Applied Biosystems, 11 December 1997.

For each PCR reaction, 8 µl (40%) of the RT reaction products (cDNA) were added to 42 µl of PCR reaction mixture containing the following: 1U AmpliTaq Gold (PE Applied Biosystems); 4 mM $MgCl_2$; 600 nM forward primer; 400 nM reverse primer; 200 uM of each dNTP, A, C, G, T; 100 nM fluorogenic probe. Reactions were hot started by incubation for 10 minutes at 95°C. Reactions were then cycled 40 times as follows: 15 seconds at 95°, and 1 minute at 60°C.

The AW#42 plasmid (provided by Ameae M. Walker, University of California, Riverside) consists of the 823 bp pre-PRL cDNA inserted into pBR322. Ten-fold serial dilutions (range: $2.3 \times 10^2$ to $2.3 \times 10^6$ copies) of AW#42 plasmid were used in reactions to generate a standard curve of threshold cycle versus starting copy number of plasmids. Data were plotted and the equation of the line was generated using Microsoft Excel.

The relative intensity of fluorescence was taken directly from the mean channel fluorescence intensity as calculated by the Coulter XL2 software statistics. Coulter defines the mean intensity as Σ log to lin (channel number) × (counts in that channel)/area.

### Acknowledgements

We would like to thank undergraduate students Amy Reed, and Jen Frankel for their help with the preliminary experiments. Special thanks to Elaine Kunze, of the Cell Analysis Center for her expert assistance with flow cytometry and Deb Grove of the Nucleic Acid Facility for her help with the Quantitative PCR. We thank Dr. Ameae Walker for her gift of the pre-PRL cDNA plasmid.

This work was supported in part by funds from the WISE Institute (Pennsylvania State University), Howard Hughes Undergraduate Research Program (Pennsylvania State University), STIRS (Pennsylvania State University PA Space Grant Consortium), an Undergraduate Molecular Biology Fellowship Award from Pfizer Inc., to Sandra L. Urtishak, who was also supported by an Academic Excellence Scholarship from the Pennsylvania State University Schreyer Honors College. Elizabeth McKenna, was supported by a Braddock Scholarship from the Eberly College of Science of the Pennsylvania State University.

### References

Bajpai, A., Hooper, K.P. and Ebner, K.E. (1991). Interactions of antisense peptides with ovine PRL. *Biochem Biophys. Res Commun.* **180**: 1312–1317.

Ebner, K.E., Varma, S. and Bjapai A. (1989) A complementary peptide to PRL produces antibody to PRL receptors. 71st *Annual Meeting of the Endocrine Society*, Seattle WA.

Grove, D.S., Bour, B., Kacsoh, B. and Mastro, A.M. (1991). Effect of neonatal milk-PRL deprivation on the ontogeny of the immune system in the rat. *Endocrine Regulations.* **25**: 11–119.

Gunes, H. and Mastro, A.M. (1996). PRL-R gene expression in rat splenocytes and thymocytes from birth to adulthood. *Mol. Cell. Endocrinol..* **117**: 41–52.

Hoeffler, J.P., F.R. Boockfor, and L.S. Frawley. (1985). Ontogeny of prolactin cells in neonatal rats: Initial prolactin secretors also release growth hormone. *Endocrinology.* **117**: 187–195.

Horseman, N.D., Zhao, W., Montecino-Rodriguez, E., Tamaka, M., Nakashima, K., Engle, S.J., Smith, F., Markoff E. and Dorshkind, K. (1997) Defective mammoporesis, but normal hemotopiesis, in mice with a targeted description of the PRL gene. *The EMBO J.* **16**: 6926–6937.

Kacsoh, B., Z. Veress, B.E. Toth, L.M. Avery, and C.E. Grosvenor. (1993). Bioactive and immunoreactive variants of prolactin in milk and serum of lactating rats and their pups. *J. Endocrinol.* **138**: 243–257.

Kearsey, J.A. and Stadnyk, A.W. (1996). Isolation and characterization of highly purified rat intestinal intraepithelial lymphocytes. *Immunol. Methods.* **194**: 35–48.

Koh, C.Y. and Phillips, J.T. (1993). PRL-R expression by lymphoid tissues in normal and immunized rats. *Mol. and Cell. Endocrinol.* **92**: R21-R25.

Kühnlein, P., Vicente, A., Varas, A., Honig, T. and Zapata, A. (1995). T cells in fetal, neonatal, and adult rat lymphoid organs. *Develop Immunol* **4**: 181–188.

Lefrancois, L. (1994). Basic aspects of intraepithelial lymphocyte immunobiology. In *Handbook* of *Mucosal Immunology*, ed. P.L. Ogra, M.E. Lamm, J.R. McGhee, J. Mestecky, W. Strober, J. Bienenstock, 287–297.

Lyscom, N. and Brueton, M.J. (1983). The development of intraepithelial and Peyer's patch lymphocyte sub-types in the small intestine of newborn rats. *Clin. Exp. Immunol.* **54** 158–162.

Montgomery, D.W., G.K. Shen, E.D. Ulrich, L.L. Steiner, P.R. Parrish, and C.F. Zukoski. (1992). Human thymocytes express a prolactin-like messenger ribonucleic acid and synthesize bioactive prolactin-like proteins. *Endocrinology.* **131**: 3019–3026.

Mukherjee, P., Mastro, A.M. and Hymer, W.C. (1990). PRL induction of interleukin-2 receptors on rat splenic lymphocytes. *Endocrinology.* **126**: 88–94.

Nagano, M., Chastre, E., Choquet, A., Bara, J., Gepach, C. and Kelly, P.A. (1995). Expression of PRL and growth hormone receptor genes and their isoforms in the gastrointestinal tract. *Am. J. Physiol.* **268**: G431-G442.

Ormandy, C.J., Camus, A., Barra, J., Damotte, D., Lucas, B., Buteau, H., Edery, M., Brousse, N., Babinet, C., Binart, N. and Kelly, P.A. (1997) Null mutation of the PRL-R gene producer multiple reproductive defects in the mouse. *Genes & Development* **11**: 167–178.

Pellegrini, I., Lebrun, J.J. and Kelly, P.A. (1992). Expression of PRL and its receptor in human lymphoid cells. *Mol. Endocrinol.* **6**: 1023–1031.

Porter, T.E. and Frawley, L.S. (1993). Regulation of lactotrope differentiation in neonatal rats by a milk-borne peptide: A review. *Endocrine Regulations.* **27**: 125–131.

Reber, P.M. (1993). Prolactin and Immunomodulation. *Am. J. of Med.* **95**: 637–644.

Russell, D.H., Matrisian, L., Kibler, R., Larson, D.F., Poulos, B. and Magun, B.E. (1984). PRL-Rs on human lymphocytes and their modulation by cyclosporine. *Biophys. Res. Commun*, **121**: 899–906.

Sabharwal, P., R. Glaser, W. Lafuse, S. Varma, Q. Liu, S. Arkins, R. Kooijman, L. Kutz, K.W. Kelley, and W.B. Malarkey. (1992). Prolactin synthesized and secreted by human peripheral blood mononuclear cells: an autocrine growth factor for lymphoproliferation. *Proc. Natl. Acad. Sci.* USA **89**: 7713–7716.

Shah, G.N. H.E. Laird II and D.H. Russell. (1991). Identification and characterization of a prolactin-like polypeptide synthesized by mitogen-stimulated murine lymphocytes. *International Immunol.* **3**: 297–304.

Steege, J.C., Buurman, W.A. and Forget, P. (1997). The neonatal development of intrapithelial and lamina propria lymphocytes in the murine small intestine. *Develop. Immunol.* **5**: 121–128.

Stevens, F.M. and C. Shaw. (1982). Prolactin-like immunoreactivity in human small-intestinal mucosa. *Brit. Med. J. Clin. Res. Ed.* **284**: 1014–1015.

Takimoto, H., Nakamura, T., Takeuchi, M., Sumi, Y., Tanaka, T., Nomoto, K. and Yoshikai, Y. (1992). Age-associated increase in number of CD4positive CD8positive intestinal intraepithelial lymphocytes in rats. *Eur. J. Immunol.* **22**: 159–164.

Thompson, F.A., Mayrhofer, G. and Cummins, A.G. (1996). Dependence of epithelial growth of the rat small intestine on T-cell activation during weaning in the rat. *Gasteroenterology*. **111**: 37–44.

Todd, D., A.J. Singh, D.L. Greiner, J.P. Mordes, A.A. Rossini, and R. Bortell. (1999). A new isolation method for rat intraepithelial lymphocytes. *J. Immunological Methods*. **224**: 111–127.

Viselli, S.M. and Mastro, A.M. (1993). Prolactin receptors are found on heterogenous subpopulations of rat splenocytes. *Endocrinology*. **129**: 983–988.

Whitworth, N.S. and Grosvenor, C.E. (1978). Transfer of milk prolactin to the plasma of neonatal rats by intestinal absorption. *J. Endocrin.* **79**: 191–199.

